# Risk of Alzheimer’s disease in patients with the syndrome of mild cognitive impairment, amnestic type (cluster analysis)

**DOI:** 10.1192/j.eurpsy.2022.926

**Published:** 2022-09-01

**Authors:** A. Simonov, T. Klyushnik, L. Androsova, N. Mikhaylova, I. Kolykhalov

**Affiliations:** 1 Mental Health Research Centre, Department Of Medical Statistics, Moscow, Russian Federation; 2 Mental Health Research Centre, Laboratory Of Neuroimmunology, Moscow, Russian Federation; 3 Mental Health Research Centre, Department Of Geriatric Psychiatry, Moscow, Russian Federation

**Keywords:** Alzheimer’s disease, aMCI, inflammation markers, cluster analysis

## Abstract

**Introduction:**

The creation of mathematical models for determining the risk of Alzheimer’s disease in patients with mild cognitive impairment based on immune markers of blood is important for differential diagnosis of AD, assessment of the clinical state of patients at different stages of the disease, and optimization of therapy.

**Objectives:**

To assess the risk of AD in patients with amnestic type of mild cognitive impairment (aMCI) with the use of such markers of inflammation as enzymatic activity of leukocyte elastase (LE) and the functional activity of α1-proteinase inhibitor (α1-PI).

**Methods:**

The object of mathematical analysis on the basis of cluster analysis and logistic regression was the database, including the results of the above immunological parameters (enzyme activity of LE and functional activity of α1-PI.) in plasma of 78 patients with aMCI receiving outpatient treatment in the clinic of FSBSI “Mental Health Research Centre”. Among the examined patients there were 25 men and 53 women aged 44 to 89 years (69.1 ± 9.95).

**Results:**

Clustering by k-means and classification by logistic regression indicate the risk of developing AD in patients with aMCI depending on the activity of LE and α1-PI in blood plasma. The total coincidence of objects included in the clusters and in the AD risk group was 94%.

**Conclusions:**

The high coincidence of the two different methods of grouping confirms the previously stated position on the possibility of identifying patients with a high risk of AD among patients with aMCI in the activity of LE and α1-PI in the blood of patients.

**Disclosure:**

No significant relationships.

